# Brain white matter changes and their associations with non‐motor dysfunction in orthostatic hypotension in α‐synucleinopathy: A NODDI study

**DOI:** 10.1111/cns.14712

**Published:** 2024-04-14

**Authors:** Lin Lin, Peilin Huang, Yingzhe Cheng, Shaofan Jiang, Jiejun Zhang, Man Li, Jiahao Zheng, Xiaodong Pan, Yanping Wang

**Affiliations:** ^1^ Department of Neurology, Center for Cognitive Neurology Fujian Medical University Union Hospital Fuzhou City China; ^2^ Fujian Institute of Geriatrics Fujian Medical University Union Hospital Fuzhou City China; ^3^ Institute of Clinical Neurology Fujian Medical University Fuzhou City China; ^4^ Fujian Key Laboratory of Molecular Neurology Fujian Medical University Fuzhou City China; ^5^ Department of Radiology Fujian Medical University Union Hospital Fuzhou City China; ^6^ Fujian Key Laboratory of Intelligent Imaging and Precision Radiotherapy for Tumors Fujian Medical University Fuzhou City China; ^7^ Center for Geriatrics Hainan General Hospital Hainan China; ^8^ Department of Endocrinology Fujian Medical University Union Hospital Fuzhou City China

**Keywords:** NODDI, non‐motor functional impairments, orthostatic hypotension, α‐synucleinopathies

## Abstract

**Background:**

The specific non‐motor symptoms associated with α‐synucleinopathies, including orthostatic hypotension (OH), cognitive impairment, and emotional abnormalities, have been a subject of ongoing controversy over the mechanisms underlying the development of a vicious cycle among them. The distinct structural alterations in white matter (WM) in patients with α‐synucleinopathies experiencing OH, alongside their association with other non‐motor symptoms, remain unexplored. This study employs axial diffusivity and density imaging (NODDI) to investigate WM damage specific to α‐synucleinopathies with concurrent OH, delivering fresh evidence to supplement our understanding of the pathogenic mechanisms and pathological rationales behind the occurrence of a spectrum of non‐motor functional impairments in α‐synucleinopathies.

**Methods:**

This study recruited 49 individuals diagnosed with α‐synucleinopathies, stratified into an α‐OH group (*n* = 24) and an α‐NOH group (without OH, *n* = 25). Additionally, 17 healthy controls were included for supine and standing blood pressure data collection, as well as neuropsychological assessments. Magnetic resonance imaging (MRI) was utilized for the calculation of NODDI parameters, and tract‐based spatial statistics (TBSS) were employed to explore differential clusters. The fibers covered by these clusters were defined as regions of interest (ROI) for the extraction of NODDI parameter values and the analysis of their correlation with neuropsychological scores.

**Results:**

The TBSS analysis unveiled specific cerebral regions exhibiting disparities within the α‐OH group as compared to both the α‐NOH group and the healthy controls. These differences were evident in clusters that indicated a decrease in the acquisition of the neurite density index (NDI), a reduction in the orientation dispersion index (ODI), and an increase in the isotropic volume fraction (FISO) (*p* < 0.05). The extracted values from these ROIs demonstrated significant correlations with clinically assessed differences in supine and standing blood pressure, overall cognitive scores, and anxiety‐depression ratings (*p* < 0.05).

**Conclusion:**

Patients with α‐synucleinopathies experiencing OH exhibit distinctive patterns of microstructural damage in the WM as revealed by the NODDI model, and there is a correlation with the onset and progression of non‐motor functional impairments.

## Graphical abstract

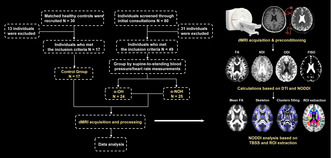

## 1 INTRODUCTION

The presence of orthostatic hypotension (OH) in α‐synucleinopathies, which encompass disorders such as Parkinson's disease (PD), dementia with Lewy bodies (DLB), and multiple system atrophy (MSA),^1^ presents a formidable therapeutic challenge and compounds prognostic concerns.^2^, ^3^, ^4^, ^5^ Currently, OH is considered to perpetuate cognitive impairment, emotional abnormalities, and other non‐motor dysfunctions^6^, ^7^, ^8^ through its impact on cerebral blood flow.^9^ Meanwhile, non‐motor dysfunctions are also considered highly disabling functional deficits.^10^ However, a comprehensive characterization of brain structural changes associated with a spectrum of non‐motor functional impairments in these patients remains unexplored. Therefore, the exploration of meaningful neuroimaging biomarkers holds promise for monitoring the progression of OH in α‐synucleinopathies, studying the relationship between cranial structures and functionality, and investigating the correlations between impaired blood pressure regulation and various aspects of non‐motor functionality.

In the early stages of α‐synucleinopathies, neurodegeneration disrupts both central and peripheral autonomic nervous networks, resulting in OH.^11^ Rapid changes in body position among OH patients with α‐synucleinopathies cause transient cerebral hypoperfusion, weakening the oxygen supply and energy provision to neural cells.^12^ This primarily manifests in the frontal region^13^ and the frontotemporal marginal system,^8^ exacerbating cognitive and psychological disorders caused by the pre‐existing neurodegenerative processes in α‐synucleinopathies.^14^ The aforementioned processes form the pathological basis for the malignant cycle of non‐motor functional impairments in α‐synucleinopathies.^15^ Early interventions in OH are crucial for reducing the risk of cognitive and emotional impairments. A large‐scale cohort study suggests that OH may not independently contribute to cognitive decline, underscoring the necessity for neuroimaging support and interpretation.^16^ Nevertheless, there is limited research exploring the correlation between OH in α‐synucleinopathies and other cognitive psychological functions with neuroimaging, which is the focus of this study.

Magnetic resonance imaging (MRI) has made significant contributions to the study of neurodegenerative diseases, providing a visual insight into macroscopic and microscopic changes in brain tissue under pathological conditions. Neurodegenerative diseases are commonly associated with gray matter, and it has been previously established that the anterior brain tissue, especially the frontal lobes, is most sensitive to cerebral hypoperfusion.^17^ However, changes in WM during the pathological process should not be overlooked, as they closely impact gray matter connections and the brain network system. For instance, in cases of cerebral hypoperfusion, damage to the conduction bundles beneath the frontal–frontal cortex connection can lead to symptoms such as executive dysfunction, attention deficits, visual–spatial impairment, and loss of interest.^18^, ^19^, ^20^, ^21^ As early as 2000, structural MRI analyses by Ballard et al. on patient populations with neurodegenerative diseases such as DLB revealed a correlation between deep WM hyperintensity and the severity of OH.^22^ Although the association between neurodegeneration and OH is gradually gaining attention, there remains a scarcity of studies that comprehensively offer a comprehensive exploration of the interplay between structural damage in WM in α‐synucleinopathies and a spectrum of non‐motor functional impairments, with OH at the forefront.

Diffusion magnetic resonance imaging (dMRI) boasts extensive research experience and a robust technical foundation. In contrast to the structural analysis of WM, dMRI, based on the diffusion of water molecules, excels in capturing microstructural changes in WM.^23^ Diffusion tensor imaging (DTI), a conventional dMRI model widely employed in the past, operates on the assumption that water diffusion conforms to a Gaussian distribution. However, it fails to directly the microscopic structure of tissue and cannot identify the cross‐sectional configuration of fiber bundles.^24^ Additionally, the single tissue compartment DTI model significantly lacks the ability to distinguish the microstructure of intracellular and extracellular spaces.^25^ In recent years, the novel model of neurite orientation dispersion and density imaging (NODDI) has gained popularity,^26^, ^27^ treating dMRI signals as biophysically meaningful parameters. NODDI offers an intuitive mapping of the microstructure of dendrites and axons, with parameters such as the neurite density index (NDI) reflecting intracellular volume fraction, the orientation dispersion index (ODI) indicating the degree of fiber direction variation, and the isotropic volume fraction (FISO) reflecting extra‐cellular isotropic diffusion.^28^, ^29^, ^30^ Studies by Kamagata et al.^31^ have confirmed that NODDI parameters can detect microstructural abnormalities in the substantia nigra and striatum in individuals with Parkinson's disease (PD) compared to healthy counterparts. Mitchell et al.^32^ found that NODDI, during longitudinal follow‐ups, revealed damage to the primary motor descending sensory tracts in PD patients. Multiple system atrophy (MSA) is characterized by reduced ODI in the putamen, decreased NDI in the cerebellar middle peduncle, and the progressive deterioration of the corticospinal tract over time.^28^ Moreover, PD patients with cognitive and psychiatric disorders exhibit widespread NDI reduction in WM compared to PD patients without non‐motor symptoms.^33^ Regarding OH, Tha et al.^34^ discovered a correlation between fractional anisotropy (FA) in the pontine tegmental area and the severity of OH in MSA patients using the DTI model. Structural MRI studies have also suggested that OH may lead to high signal intensity in WM.^22^, ^35^ However, results from a multicenter study indicated no difference in WM signals between PD and DLB patients, irrespective of the presence or absence of OH,^36^, ^37^ suggesting the need for more sophisticated dMRI models in subsequent studies. In the context of α‐synucleinopathies, there is a scarcity of research employing the NODDI model to synthesize assessments of WM damage in OH patients, with insufficient attention directed towards elucidating the correlation of cognitive and emotional symptoms in OH with combined WM damage. Therefore, this study aims to undertake meticulous high‐precision data collection using dMRI techniques and NODDI model research based on the α‐synucleinopathies registry study (NCT05527067). This endeavor aspires to assist in unraveling the microstructural changes in the brain's WM associated with a spectrum of non‐motor functional disorders, primarily centered around OH.

Expanding on the previously mentioned, this research postulates that the dMRI technology utilizing the NODDI model will unveil distinct locations of WM damage within the brain in individuals with OH in the context of α‐synucleinopathies, as compared to those without OH. These identified regions are presumed to play a role in the onset or progression of OH. Simultaneously, there is a hypothesis that the observed disparities in brain regions may correlate with other non‐motor functional disorders, including cognitive impairment and anxiety‐depressive states. The elucidation presented offers novel evidence for the supplementation of pathogenic mechanisms and pathological explanations of a spectrum of non‐motor functional disorders in α‐synucleinopathies. Additionally, it functions as a neuroimaging biomarker for the early detection of critical complications in α‐synucleinopathies.

## 2 METHODS

### 2.1 Ethics and informed consent

The ethical approval for this initiative was granted by the Ethics Committee of FJMUUH (2020YF004‐01). The study adhered to the guidelines outlined in the Cohort‐PDS cohort (NCT05527067, https://clinicaltrials.gov). The entire undertaking was conducted in strict accordance with the principles set forth in the Declaration of Helsinki (1975) and the National Statement on Ethical Conduct in Research Involving Humans (1999). No conflicts of interest were present among any of the authors involved in this study.

### 2.2 Participants

**FIGURE 1:**
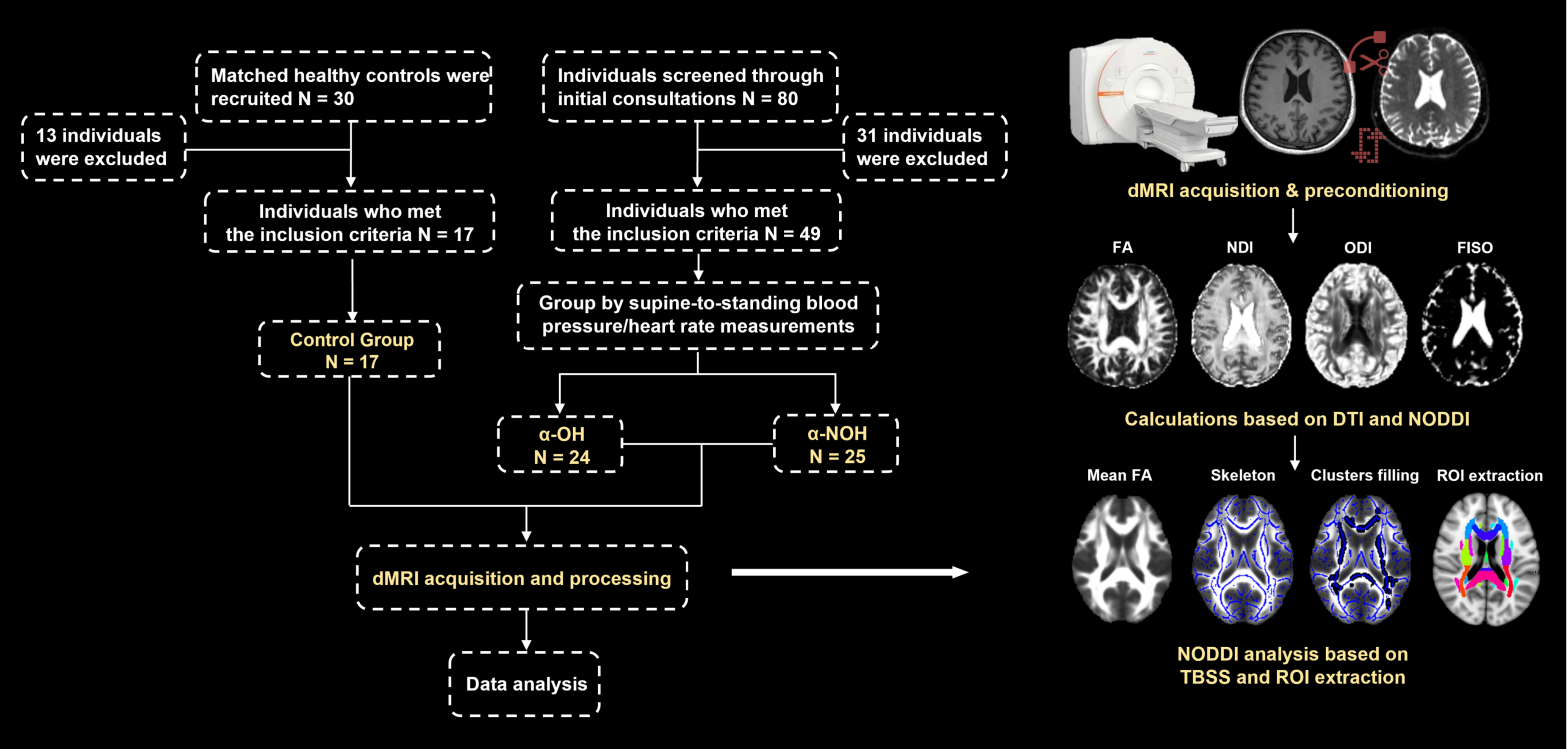
Flowchart of participant enrollment and imaging data collection and analysis.

The enrollment process is depicted in Figure 1. For the duration of this investigation, patients were consecutively enrolled at Fujian Medical University Union Hospital (FJMUUH) spanning the period from September 2021 to June 2023. Inclusion criteria involved the diagnosis of PD, MSA, and DLB, adhering to the clinical diagnostic criteria for PD established by the Movement Disorder Society in 2015,^38^ the second consensus statement on the diagnosis of MSA in 2008,^39^ and diagnosis and management of DLB in 2017^40^; and signature of informed consent forms by participants. Exclusion criteria encompassed contraindications to MRI examinations, internal metal content, claustrophobia, pregnancy, fever, and inability to cooperate. The diagnostic assessments for inclusion were overseen by a seasoned clinical physician with two decades of experience in the diagnosis and treatment of neurodegenerative diseases. Following the screening process, 49 patients diagnosed with α‐synucleinopathies were incorporated into the study, comprising 36 PD cases, 11 MSA cases, and 2 DLB cases.

### 2.3 The control group

A control group consisting of individuals in good health was incorporated, with careful consideration given to matching their gender and age with those of the patients. The eligibility criteria are outlined as follows: individuals of either gender, aged between 35 and 80 years, demonstrating a willingness to collaborate with the study, and consenting to sign informed consent forms. Exclusion criteria included the presence of neurological disorders and contraindications to MRI examinations. Ultimately, 17 healthy controls were incorporated in the study (Figure 1).

### 2.4 Acquisition and grouping of orthostatic HR‐BP data

All patients maintained a supine position in a designated, independent, and tranquil enclosed evaluation room for 20 min preceding the measurement of their blood pressure (BP) and heart rate (HR). Automated equipment (Omron‐HEM‐907 XL; Omron Healthcare) was employed for the collection of upper arm BP and HR (pulse rate) readings. Concurrently, the mean arterial pressure (MAP) was computed. Subsequently, patients were instructed to stand up briskly, unaided, with BP and HR measurements taken at 0, 1, 3, and 5 min post‐standing, respectively. Discrepancies in HR, MAP, systolic blood pressure (SBP), and diastolic blood pressure (DBP) at 0, 1, 3, and 5 min post‐standing in comparison to the supine position were calculated. These variations were denoted as ΔHR, ΔMAP, ΔSBP, and ΔDBP, respectively (negative values indicating a decrease in HR or BP relative to the supine position).

Employing stringent BP measurement criteria and adhering to the established OH diagnostic criteria, patients were categorized into the α‐OH group (*N* = 24) and the α‐NOH group (*N* = 25).^41^

### 2.5 Neuropsychological Assessment

The overall cognitive function assessment involved the application of the mini‐mental state examination (MMSE),^42^ Montreal Cognitive Assessment (MOCA),^43^ and Addenbrooke's Cognitive Examination version III (ACE‐III) scales.^44^ The emotional evaluation included the 14‐item Hamilton Anxiety Rating Scale (HAMA)^45^ and the 24‐item Hamilton Depression Rating Scale (HAMD).^46^ The administration of these neuropsychological assessment scales was conducted by the same neurologist with over 5 years of clinical experience and specialized training in professional evaluation.

### 2.6 dMRI scan parameter settings

dMRI data acquisition was conducted on a 3 T magnetic resonance scanner equipped with a 64‐channel receive‐only head coil (MAGNETOM Prisma; Siemens Healthcare, Erlangen, Germany). The participants' heads were immobilized and they were instructed to avoid head movement. The *B* value was configured at 0, 1000, 2000, and 3000 s/mm^2^ (scanned in 95 directions), employing 72 slices with a thickness of 2 mm. Additional scan parameters included a repetition time (TR) of 5800 ms, time to echo (TE) of 91 ms, field‐of‐view (FOV) of 215 × 215 mm^2^, generalized autocalibrating partially parallel acquisition of 2, slice acceleration factor of 2, number of averages of 1, voxel size of 2 × 2 × 2 mm^3^ without gap, and an acquisition time of 9 min and 44 s. High‐resolution T1‐weighted images were acquired using a Magnetization Prepared‐Rapid Gradient Echo (Mprage) sequence with the following parameters: TR of 2300 ms, TE of 2.32 ms, FOV of 240 × 240 mm^2^, number of averages of 1, voxel size of 0.9 × 0.9 × 0.9 mm^3^, 192 slices, and an acquisition time of 4 min and 44 s.

### 2.7 dMRI data preprocessing and computation

The image processing is illustrated in Figure 1. dMRI scan images of both patients and healthy controls included in the study underwent rigorous screening, involving the validation of consistent scan parameters and the exclusion of images containing artifacts, noise, or head motion. The selected images underwent preprocessing of raw files and computation of specific indices. Initially, the raw DICOM files were imported into the MRicron software package (https://www.nitrc.org/projects/mricron) using the dcm2nii routine, converting them into NIFTI files and organizing them into respective groups. Subsequently, the FMRIB's Diffusion Toolbox (FSL‐FDT; part of FSL version 5.0, http://fsl.fmrib.ox.ac.uk/fsl/fslwiki/FDT) was utilized for correcting motion eddy current correction and gradient directions.^47^ The resulting skull‐stripped b0 image was used as a brain mask. The FSL dtifit command was employed to compute the Fractional Anisotropy (FA) index based on the DTI model. The acquisition of three NODDI indices (NDI, ODI, FISO) scalar index via the dtifit function in FSL.

### 2.8 Comparison of voxel‐wise differences based on DTI and NODDI models

As illustrated in Figure 1, tract‐based spatial statistics (TBSS, part of FSL version 5.0, http://fsl.fmrib.ox.ac.uk/fsl/fslwiki/TBSS/UserGuide), was employed for WM difference analysis.^47^ The process unfolded as follows: The FA files were registered to the 1 × 1 × 1 mm Montreal Neurological Institute (MNI) standard space using the FMRIB58_FA template (http://www.fmrib.ox.ac.uk/fsl/data/FMRIB58_FA).^48^, ^49^ After setting an FA threshold greater than 0.2, filtering, and binarization, a group‐average FA skeleton file was generated. Subsequently, individual FA files were projected onto the average FA skeleton. A parallel process was applied to project NODDI indices (NDI, ODI, FISO) onto the standardized FA skeleton. Voxel‐wise statistics involved non‐parametric testing coupled with threshold‐free cluster enhancement (TFCE), a method for cluster‐level correction. A total of 5000 permutations were conducted, inflating results for *p* < 0.05.

For the presentation of differences in specific brain regions, we referenced the JHU fiber tractography atlas as an anatomical basis.^50^ JHU regions with statistically significant differences and research significance were designated as regions of interest (ROI). Subsequently, NODDI index parameters for the key ROIs, including NDI, ODI, and FISO, were extracted and averaged.

### 2.9 Statistical analysis

Whether data followed a normal distribution was assessed through the Shapiro‐Wilks *W* test. In cases where the data deviated from a normal distribution, non‐parametric tests were employed. Multiple testing was conducted using Kruskal‐Wallis followed by Bonferroni posthoc analysis. Categorical variables were analyzed using the chi‐square test. Spearman's correlation was applied for non‐normally distributed data and multiple comparisons were performed for correction. Statistical analysis was carried out utilizing the SPSS software (Version 26.0; Chicago, Illinois, USA). Graphical representations were generated using the R software (version 4.2.3; R Core Team, New Zealand, http://www.r‐project.org/).

## 3 RESULTS

### 3.1 Baseline demographics, blood pressure measurements, and neuropsychological test scores: group‐wise comparison

Table 1 presents the demographic information for the α‐OH group (*n* = 24), α‐NOH group (*n* = 25), and control group (*n* = 17). The table indicates no statistically significant differences (*p* > 0.05) in gender, age, duration of illness, education level, and Body Mass Index (BMI) among the groups. Analysis of supine‐to‐standing blood pressure within 5 min revealed that ΔMAP, ΔSBP, and ΔDB in the α‐OH group were significantly lower than those in the α‐NOH group and control group (*p* < 0.05), suggesting a greater drop in blood pressure in the supine position for the α‐OH group. There were no significant differences in the scores of the three cognitive assessments between the α‐OH and α‐NOH groups (*p* > 0.05). However, the α‐OH group scored lower than the control group in the ACE‐III assessment, which has the most items (*p* < 0.05). Additionally, the α‐OH group exhibited higher scores in HAMA‐14 and HAMD‐24 compared to the α‐NOH and control groups (*p* < 0.05), indicating a notable presence of anxiety and depressive states in the α‐OH group.

**TABLE 1:** Inter‐group comparison of demographic data, supine‐standing blood pressure changes, cognitive levels, and psychological status across the three groups.

	α‐OH (*N* = 24)	α‐NOH (*N* = 25)	Ctrl (*N* = 17)	Comparison	*p*‐Value
Gender (male, *n*)	16	10	7	–	0.123
Age, y	63.5 (57.5, 70.0)	63.0 (59.0, 69.0)	60.0 (56.0, 67.0)	–	0.636
Duration of illness, y	3.0 (2.0, 4.0)	4.0 (3.0, 5.0)	–	–	0.061
Education, y	8.5 (6.0, 12.0)	9.0 (6.0, 11.0)	9.0 (5.0, 10.5)	–	0.877
BMI, kg/m^2^	22.8 (21.3, 24.6)	24.5 (21.5, 25.3)	24.4 (22.0, 26.3)	–	0.359
ΔMAP – 0 min, mmHg	−11.8 (−17.0, −9.25)	1.0 (−2.7, 6.5)	3.7 (0.5, 7.5)	α‐OH < α‐NOH, α‐OH < Ctrl	<0.001
ΔMAP – 1 min, mmHg	−9.3 (−14.6, −2.5)	2.7 (−1.5, 6.5)	3.7 (0.5, 7.2)	α‐OH < α‐NOH, α‐OH < Ctrl	<0.001
ΔMAP – 3 min, mmHg	−7.5 (−11.3, −5.3)	−0.7 (−3.0, 5.7)	4.0 (−0.2, 7.7)	α‐OH < α‐NOH, α‐OH < Ctrl	<0.001
ΔMAP – 5 min, mmHg	−7.5 (−12.2, −4.1)	1.7 (−2.5, 7.2)	2.0 (−0.5,8.2)	α‐OH < α‐NOH, α‐OH < Ctrl	<0.001
ΔSBP – 0 min, mmHg	−21.0 (−27.5, −20.3)	0 (−7.0, 6.5)	3.0 (−4.5, 9.0)	α‐OH < α‐NOH, α‐OH < Ctrl	<0.001
ΔSBP – 1 min, mmHg	−18.0 (−24.0, −11.5)	1.0 (−3.5, 6.5)	3.0 (−5.5, 12.5)	α‐OH < α‐NOH, α‐OH < Ctrl	<0.001
ΔSBP – 3 min, mmHg	−15.5 (−25.8, −11.3)	−2.0 (−6.5, 6.0)	1.0 (−4.0, 7.5)	α‐OH < α‐NOH, α‐OH < Ctrl	<0.001
ΔSBP – 5 min, mmHg	−14.5 (−27.5, −10.3)	−3.0 (−6.0, 8.5)	0 (−4.0, 6.0)	α‐OH < α‐NOH, α‐OH < Ctrl	<0.001
ΔDBP – 0 min, mmHg	−7.0 (−12.8, −3.5)	1.0 (−2.0, 7.5)	4.0 (1.5, 8.5)	α‐OH < α‐NOH, α‐OH < Ctrl	<0.001
ΔDBP – 1 min, mmHg	−6.0 (−10.8, 3.3)	4.0 (−2.0, 6.5)	5.0 (3.0, 6.0)	α‐OH < α‐NOH, α‐OH < Ctrl	0.001
ΔDBP – 3 min, mmHg	−4.5 (−8.5, −2.0)	1.0 (−3.5, 7.0)	6.0 (1.0, 8.0)	α‐OH < α‐NOH, α‐OH < Ctrl	<0.001
ΔDBP – 5 min, mmHg	−2.5 (−9.3, −0.3)	1.0 (−2.0, 8.5)	2.0 (0, 10.5)	α‐OH < α‐NOH, α‐OH < Ctrl	<0.001
MOCA	22.0 (18.3, 25.8)	23.0 (22.0, 28.0)	25.0 (23.0, 28.0)	–	0.102
MMSE	24.5 (20.0, 28.8)	27.0 (23.5, 29.0)	28.0 (26.5, 30.0)	–	0.103
ACE‐III	71.0 (56.8, 88.0)	80.0 (74.0, 88.0)	85.0 (83.0, 92.5)	α‐OH < Ctrl	0.002
HAMA	10.5 (7.3, 14.8)	6.0 (3.0, 10.0)	5.0 (2.5, 6.0)	Ctrl < α‐OH, α‐NOH < α‐OH	<0.001
HAMD	12.0 (6.0, 16.8)	4.0 (0, 9.0)	2.0 (0.5, 5.0)	Ctrl < α‐OH, α‐NOH < α‐OH	<0.001

*Note*: Continuous variables were described using median (IQR) for non‐parametric data in the tables.

Abbreviations: Ctrl, control group; y, years.

### 3.2 Correlation analysis of supine‐to‐standing blood pressure changes with cognitive and psychological states

**FIGURE 2:**
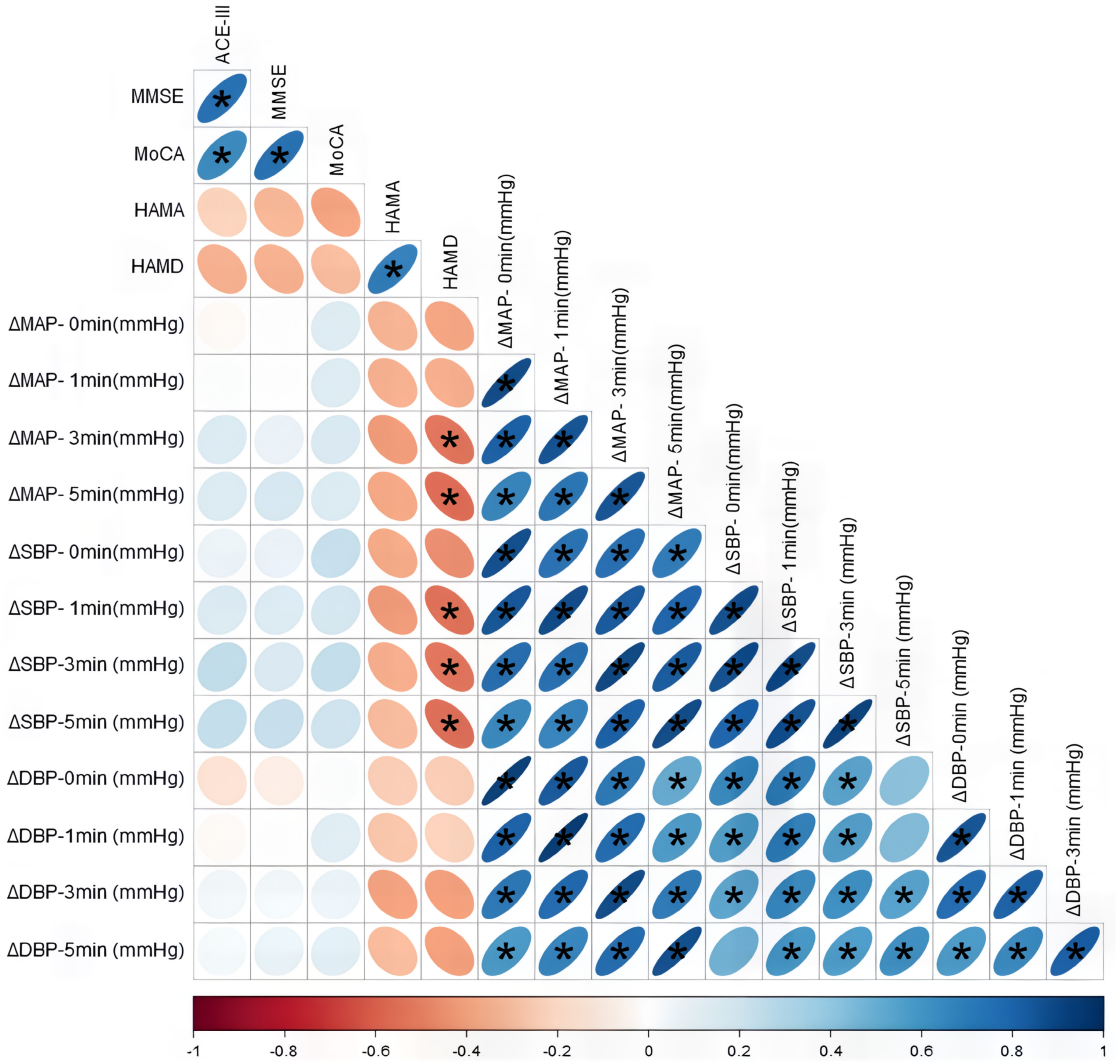
Correlation heatmap between supine‐standing blood pressure change values and neuropsychological assessment scores. *p < 0.05. The flatter the graph, the darker the color, indicating a stronger correlation.

To assess the potential correlation between the severity of OH and cognitive and psychological states, Spearman's correlation was employed to examine the supine‐to‐standing blood pressure differences in α‐synucleinopathies patients in relation to various neuropsychological assessment scores (Figure 2). The findings revealed a significant association between more pronounced drops in standing blood pressure and increased severity of depression. Specifically, the magnitude of blood pressure reduction within the initial 5 min of standing showed a moderate correlation with HAMD‐24 scores (*p* < 0.05). However, the degree of blood pressure drop did not show a direct correlation with overall cognitive function (*p* > 0.05).

### 3.3 TBSS Analysis based on the NODDI Model

**FIGURE 3:**
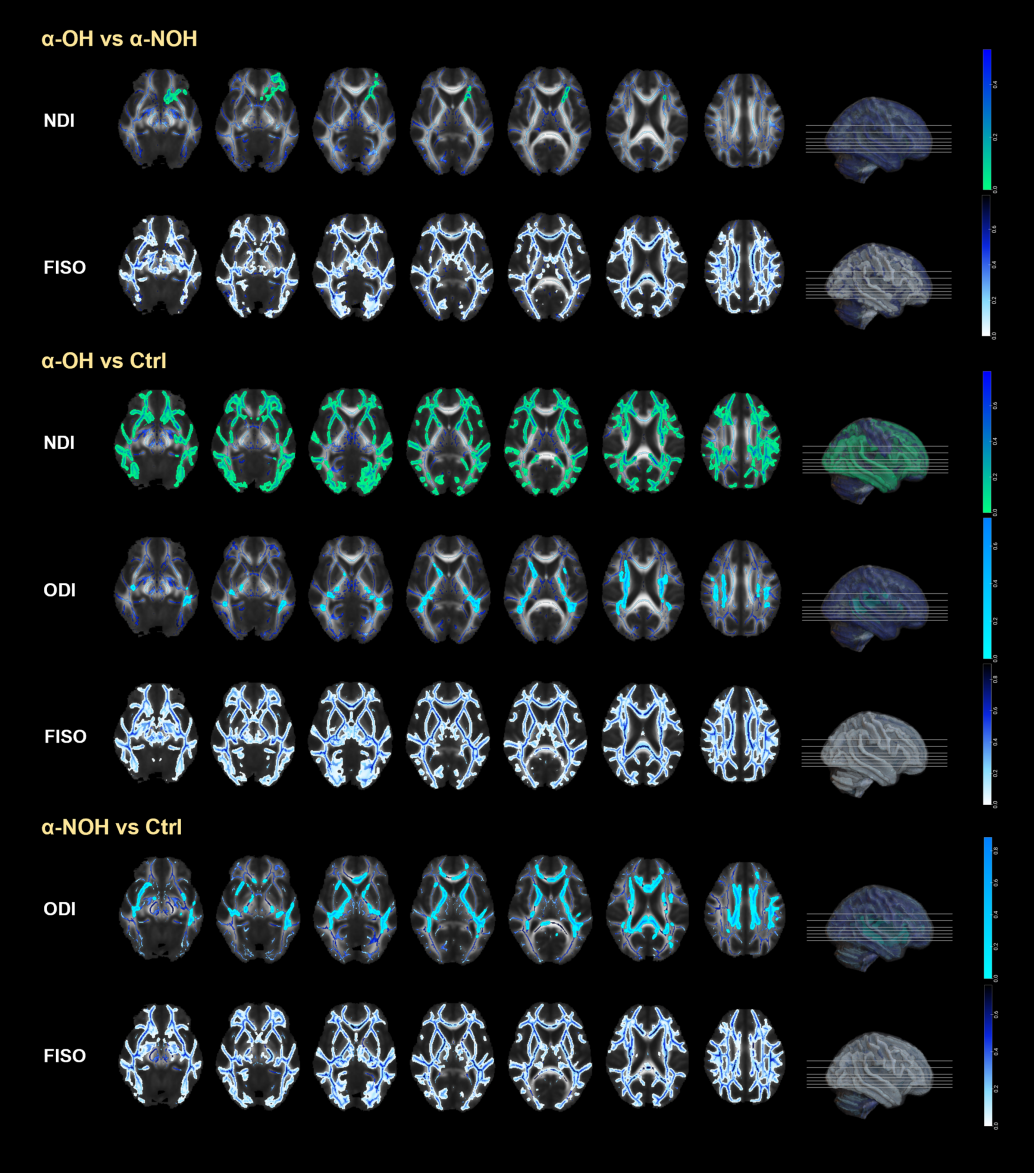
Inter‐group TBSS analysis results based on the NODDI model (schematic diagram composed of the mean_FA file overlaid with WM skeleton and expanded results of differential clusters).

In the comparison among the three groups, pairwise TBSS analyses were conducted for the NDI, ODI, and FISO parameters derived from the NODDI model, with the expanded differential brain regions illustrated in Figure 3. Notably, there was a distinct cluster of differences in NDI comparison between the α‐OH and α‐NOH groups, localized in the anterior part of the dominant hemisphere (α‐OH < α‐NOH, *p* < 0.05). This cluster predominantly involved the WM connected to the left frontal lobe, encompassing the left inferior fronto‐occipital fasciculus, left anterior thalamic radiation, left uncinate fasciculus, forceps minor, left superior longitudinal fasciculus, left superior longitudinal fasciculus (temporal part), left cingulum (cingulate gyrus), and left corticospinal tract, as well as the right uncinate fasciculus (Table 2). Remarkably, NDI exhibited a more widespread reduction in the α‐OH group compared to the control group (*p* < 0.05, Table S2), while no significant differences were observed between the α‐NOH and control groups (*p* > 0.05). The differences in ODI were evident in that both α‐synucleinopathies groups demonstrated lower values compared to the control group (*p* < 0.05, Tables S3 and S5), predominantly concentrated in deep WM adjacent to the lateral ventricles. Additionally, FISO demonstrated extensive differences across the whole brain through the pairwise comparison among the three groups (α‐OH > α‐NOH > control group, *p* < 0.05, Tables S1, S4, S6).

**TABLE 2:** TBSS analysis results of α‐OH and α‐NOH groups based on NDI parameters.

Regions & proportion, %	Cluster voxels	MNI‐peak coordinates			*p*‐Value
		*X* (mm)	*Y* (mm)	*Z* (mm)	
Inferior fronto‐occipital fasciculus L: 7.40616	1332	−11	18	−11	0.044
Anterior thalamic radiation L: 6.28979					
Uncinate fasciculus L: 4.86787					
Forceps minor: 0.46021					
Superior longitudinal fasciculus L: 0.148649					
Superior longitudinal fasciculus (temporal part) L: 0.121622					
Cingulum (cingulate gyrus) L: 0.0975976					
Corticospinal tract L: 0.00225225					
Uncinate fasciculus R: 0.00225225					

*Note*: All the above fiber bundles were from the same cluster.

Abbreviations: L, left‐lateralized fiber; MNI‐peak coordinates, peak locations are expressed in coordinates according to MNI space; R, right‐lateralized fiber.

### 3.4 ROI extraction analysis based on the NODDI model

**FIGURE 4:**
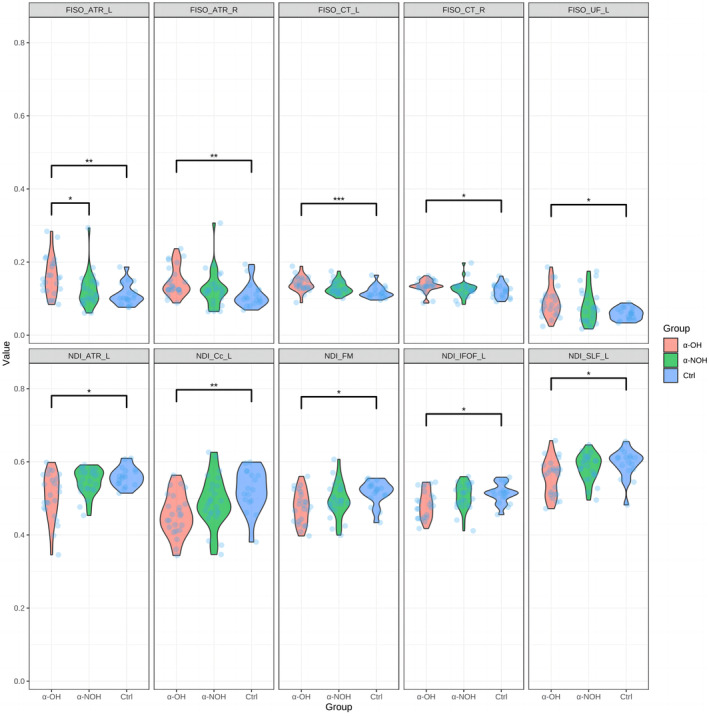
Violin plots of non‐parametric tests for ROI extraction based on the NODDI model in three groups. *p < 0.05; **p < 0.01; ***p < 0.001. ATR_L, left anterior thalamic radiation; ATR_R, right anterior thalamic radiation; Cc_L, left cingulum (cingulate gyrus); CT_L, left corticospinal tract; CT_R, right corticospinal tract; FM, forceps minor; IFOF_L, left inferior fronto‐occipital fasciculus; SLF_L, left superior longitudinal fasciculus; UF_L, left uncinate fasciculus.

Based on the TBSS analysis in the α‐OH and α‐NOH groups, the fiber bundles involved in the differential clusters under the NDI parameters were taken as key ROIs for the value extraction of NDI parameters, and those involved in the differential clusters under the FISO parameters were taken as key ROIs for the value extraction of FISO parameters (Table 1 and Table S1). Non‐parametric testing was employed, with the results depicted in Figure 4. Notably, only the FISO values of the left anterior thalamic radiation exhibited differences between the α‐OH and α‐NOH groups, which were greater in the α‐OH group than in both the α‐NOH group (*p* < 0.05) and the control group. Among the remaining indices, several fiber bundles predominantly located in the bilateral hemisphere (namely the right anterior thalamic radiation, bilateral corticospinal tract, and left uncinate fasciculus) showed differences in FISO values, which were greater in the α‐OH group than in the control group. Some (namely the left anterior thalamic radiation, left cingulum (cingulate gyrus), forceps minor, left inferior fronto‐occipital fasciculus, and left superior longitudinal fasciculus) exhibited lower NDI values in the α‐OH group than in the control group (*p* < 0.05).

### 3.5 Correlation analysis between quantitative values of interest fiber bundles and severity of non‐motor symptoms in α‐synucleinopathies

**FIGURE 5:**
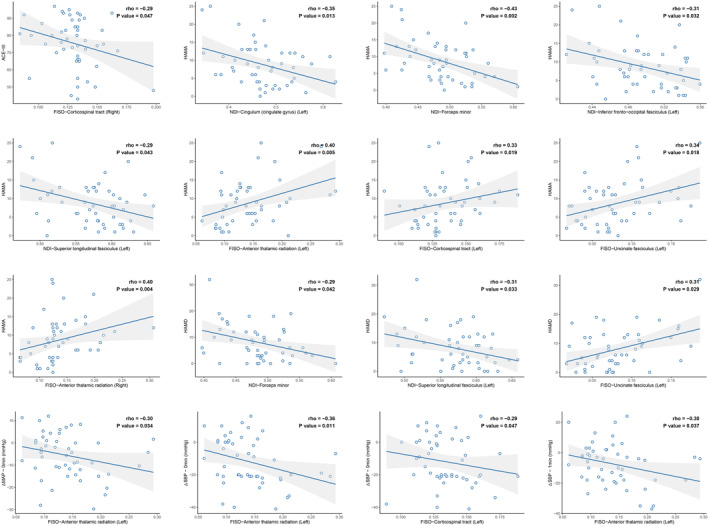
Correlation analysis results of NODDI model parameters with supine‐standing blood pressure change values and neuropsychological assessment scores.

In the α‐synucleinopathies participants, following the extraction of NODDI parameter values for the specified fiber bundles, Spearman's correlation was conducted between the NDI and FISO results of all ROIs and the changes in supine‐to‐standing blood pressure, as well as the outcomes of neuropsychological assessments, individually (Figure 5). The findings revealed that the extent of blood pressure drop, as represented by ΔMAP and ΔSBP within the first 5 min of the supine‐to‐standing transition, was primarily correlated with the FISO values of left anterior thalamic radiation and corticospinal tract (*p* < 0.05). Concerning overall cognitive levels, higher FISO values of the right corticospinal tract correlated negatively with elevated ACE‐III scores, indicating superior cognitive performance. Regarding the psychological assessment of the participants with α‐synucleinopathies, lower NDI values of the left cingulum (cingulate gyrus), forceps minor, left inferior fronto‐occipital fasciculus and left superior longitudinal fasciculus, and higher FISO values of the left anterior thalamic radiation, left corticospinal tract, left uncinate fasciculus and right anterior thalamic radiation were associated with more pronounced anxiety (HAMA‐14); lower NDI values of the forceps minor and left superior longitudinal fasciculus, and higher FISO values of the left uncinate fasciculus were associated with more pronounced depression (HAMD‐24).

## 4 DISCUSSION

This study, based on the NODDI model parameters using the novel high‐precision dMRI, employs a comprehensive neuroimaging quantification approach that integrates TBSS with ROI extraction. It aims to describe the WM imaging features in α‐synucleinopathies patients who experience OH, identify differences in WM microstructure compared to those without OH and further, and subsequently explore the correlation between WM damage and abnormal blood pressure fluctuations, cognitive impairment, and anxiety‐depression states in participants. The goal is to provide new evidence and identify potential novel imaging biomarkers for the pathological mechanisms underlying a spectrum of non‐motor functional impairments in α‐synucleinopathies patients experiencing OH.

Non‐motor symptoms specific to α‐synucleinopathies pose a high risk of disability, involving the dysregulation of the autonomic nervous system, cognitive abnormalities, and mood disorders. While previous research has shed light on the pathogenesis of non‐motor symptoms, discussions on the interplay between several symptoms have been limited. Particularly, when autonomic nervous system impairment affects blood pressure regulation, significant fluctuations in cerebral perfusion may lead to a cascade of associated brain parenchymal damage, potentially exacerbating cognitive and emotional disturbances. Longitudinal studies have confirmed sustained cognitive decline^7^ and mood disturbances^8^ in the presence of chronic OH. In our study, among α‐synucleinopathies patients experiencing OH, a more pronounced blood pressure decline during postural changes was associated with more evident mood disorders, particularly depressive states, aligning with previous research findings.^8^ Importantly, we did not observe a direct correlation between OH and overall cognition. It is speculated that rapid blood pressure fluctuations may predominantly impact cognitive levels during positional changes, rather than directly influencing cognitive function during periods of quiet stability. Further neuroimaging discoveries revealed more extensive signs of brain damage in the α‐OH group compared to the α‐NOH group. The extracted values of fiber bundles within this region displayed associations with the severity of OH, cognitive levels, and the degree of anxiety and depression. This suggests that the damage in this area might contribute to non‐motor functional impairments, and OH, by compromising brain tissue, may further propel the progression of other non‐motor symptoms. This provides a plausible explanation for its impact on overall cognitive levels. Despite the lack of apparent cognitive impairment in commonly used screening scales (MMSE and MOCA) among the α‐OH group, which necessitates identification by ACE‐III with more comprehensive assessment items, cognitive levels of α‐synucleinopathies participants still exhibited a correlation with the magnitude of WM damage parameters. This may signify an early preclinical state of cognitive impairment, with imaging manifestations potentially serving as early warning biomarkers.

The distinctive parameters of the NODDI model play a crucial role in deducing the specific microstructural damage occurring in the brain WM of the participants in the α‐OH group, in conjunction with TBSS to identify differential brain regions. Notably, FISO exhibits extensive differences in brain regions across α‐synucleinopathies populations relative to the control group, as well as between the α‐OH and α‐NOH groups. All patients with α‐synucleinopathies manifest widespread structural loosening of fiber tissues and increased interstitial water molecules relative to the control group, corresponding to diverse neurological functional impairments.^51^ In the context of matched demographic information and disease duration, both the α‐OH and α‐NOH groups demonstrate widespread increases in FISO, suggesting that inadequate blood perfusion itself may exacerbate neuroinflammation or degeneration in α‐synucleinopathies. However, NDI values only reveal differential brain regions in the α‐OH group compared to both α‐NOH or the control group. Combining past research on the attention to NDI parameters in α‐synucleinopathies,^33^, ^52^ this study suggests widespread axonal damage and subsequent demyelination in the α‐OH group compared to the control group; and meanwhile, relative to the α‐NOH group, localized WM damage beneath the frontal and temporal cortices of the dominant hemisphere is observed, leading to an increase in axonal extracellular space. Drawing on the pathology of the primary disease, it can be speculated that the aggregation of α‐syn in axons triggers axonal degeneration, hindering axonal transport function.^33^ Simultaneously, the low perfusion effect combined with OH results in selective oligodendrocyte death, resulting in demyelinating fiber degeneration.^53^ However, these manifestations were not observed when compared to healthy individuals, suggesting that significant blood pressure fluctuations likely impact the process of the axonal microstructure repair and regeneration in WM. ODI parameters are sensitive to the characterization of microstructural information in crossing fibers and are susceptible to changes in axons.^54^ In this study, while no differential clusters of ODI were identified between the two groups of α‐synucleinopathies, a decrease in deep WM regions relative to the control group was observed. It is speculated that there may be selective axonal degeneration and loss in densely crossed regions of the deep nuclei tracts in α‐synucleinopathies patients, thereby enhancing the consistency of neuronal arrangement.^55^

Leveraging parameters within the NODDI model, this study pinpointed identified several fiber bundles closely associated with non‐cognitive symptoms in the α‐OH group. After localizing target clusters using TBSS, the analysis of the numerical values extracted from the designated ROIs was conducted. This dual‐pronged approach, amalgamating two precise and synergistic methods, yields pivotal insights upon cross‐referencing with clinical data, shedding light on the fiber bundles integral to the progression of non‐motor symptoms. Initially, in non‐parametric inter‐group analyses based on ROIs, the α‐OH group exhibited increased FISO in the left anterior thalamic radiation relative to the α‐NOH group. The decrease in fiber tissue density and complexity implies potential inflammatory reactions, given that this fiber bundle is a key connection in the prefrontal cortex‐thalamus loop involving the anterior thalamic nucleus and dorsomedial nucleus.^56^ This disruption may be attributed to the direct impact of α‐synuclein deposition, leading to the disturbance of autonomic signals in the prefrontal lobe,^57^ aggravating OH. Additionally, under the influence of OH, ischemic inflammatory damage further exacerbates the situation, creating a detrimental cycle. The aforementioned pathology also disrupts the emotional regulation function of the anterior thalamic radiation,^58^ explaining evident blood pressure‐related depression observed in α‐synucleinopathies participants. This fiber bundle is also implicated in the control of divergent thinking, creativity, and memory,^59^, ^60^ potentially elucidating the poorer performance of the α‐OH group in ACE‐III, which assesses more comprehensive cognitive domains, in comparison to the control group. Upon associating NODDI indices with clinical data in α‐synucleinopathies participants, attention was drawn to the corticospinal tracts, showing correlations with the extent of blood pressure fluctuations and cognitive levels. It is speculated that neuroinflammation, edema, and decreased fiber complexity may be associated with insufficient cerebral perfusion. Previous research has confirmed damage to the corticospinal tract in longitudinal follow‐ups of patients with PD and MSA. This finding has also been acknowledged in early pathological research.^61^, ^62^ Corticospinal projection neurons are the main subcerebral projection neurons of layer V neurons in the cerebral cortex.^63^, ^64^ Interestingly, a study on umbilical cord blood transplantation therapy for neonatal brain disease found that the improvement in corticospinal tract development was closely related to the enhancement of cognitive function,^65^ demonstrating the importance of this fiber bundle in regulating cognitive function. Since the corticospinal tract is involved in the most important motor control, this may also lead to impairments in executive function and other complex cognitive assessments, including, including visual–spatial abilities and other motor‐cognitive composite abilities, such as eye‐hand coordination,^66^ thus closely correlating fiber tract damage with reduced ACE‐III scores. Additionally, left anterior thalamic radiation exhibits simultaneous correlations with OH and emotional states. Specifically, the severity of OH is related to the sparsity in the distribution of fiber tissue observed in FISO of the left anterior thalamic radiation. The mechanisms underlying the impact of the left anterior thalamic radiation on psychological states involve low perfusion‐induced axonal degeneration or loss and reduced complexity of neural networks affecting its connections and regulation of the mediodorsal thalamus and frontal cortex, both of which are related to emotional processing.^67^, ^68^, ^69^, ^70^ As an important thalamus‐related connection, damage to the anterior thalamic radiation is considered a marker of high anxiety. An interesting study on psychological disorders found that the more severe the damage to the anterior thalamic radiation, the stronger the empathy and sympathy, resulting in negative emotions.^71^ Combining single‐photon emission computed tomography findings, PD patients with mood disorder exhibit insufficient blood flow in the frontal‐temporal‐marginal system,^8^ and most of anterior thalamic radiation which originates in the frontal lobe, may have experienced secondary damage to conduction bundles, supporting the hypothesis in this study that OH, due to insufficient cerebral blood flow, leads to secondary emotional disturbances. The significant anxiety and depression observed in α‐synucleinopathies participants in this study were considered to be closely correlated with widespread fiber bundle inflammation and degeneration accompanied by axonal demyelination, etc., in the imaging analysis. Among them, the forceps minor, left superior longitudinal fasciculus, and left uncinate fasciculus are speculated to be involved in the mechanism of anxiety and depression simultaneously. Forceps minor has been reported to be associated with impulsive personality, antisocial behavior, and decreased reward‐punishment capabilities,^72^, ^73^, ^74^ while damage changes in the left superior longitudinal fasciculus and left uncinate fasciculus in the DTI model of anxiety and depression patients have been confirmed in previous clinical studies,^75^ especially in the long contact fibers in the dominant hemisphere of PD patients with mood disorders.^76^ In conclusion, this study identified specific WM damage in the cerebral hemispheres of OH patients with α‐synucleinopathies, supplementing the previously predominant view of brainstem damage primarily managing the autonomic nervous system.^77^ WM associated with blood pressure regulation disruption highly overlaps with WM related to cognition and emotions, suggesting that OH may disrupt WM fiber bundles connected to the higher cortex, subsequently affecting corresponding cognitive and emotional regulation functions. It can also be inferred that damage to fiber bundles conducting and managing the autonomic nervous system exacerbates the severity of OH, forming a vicious cycle.

This study reveals imaging alterations in brain WM in the context of α‐synucleinopathies with OH and explores their interconnections with non‐cognitive functions. We propose that, compared to healthy individuals, the widespread deposition of early‐stage α‐syn in the primary disease mediates the occurrence of non‐motor functional symptoms. Simultaneously, OH patients with matched disease progression exhibit extensive inflammation, edema, and tissue rarefaction compared to those without OH; and also manifest damage or even loss of axons in WM of the anterior dominant hemisphere. The OH‐specific brain damage mentioned above may contribute to the onset and progression of OH and, alternatively, the low perfusion damage associated with OH may be involved in influencing the occurrence of cognitive impairment and emotional disturbances. However, the study results do not support a causal relationship. Moreover, the significance of the NODDI model extends to the early detection of non‐motor functional abnormalities in α‐synucleinopathies, with manifestations of WM damage related to cognition even in the pre‐early stages of cognitive impairment.

## 5 CONCLUSION

Patients with α‐synucleinopathies experiencing OH exhibit unique imaging features of microstructural WM damage under the NODDI model. These findings correlate with the onset and progression of non‐motor functional impairments.

## AUTHOR CONTRIBUTIONS

All authors contributed to this study. Y.P.W. and X.D.P. provided overall guidance for the study. L.L., P.L.H., Y.Z.C., and S.F.J were involved in the collection of cases and observation parameters. L.L. performed the data analysis and manuscript editing. J.J.Z., M.L., and J.H.Z. assisted in data verification. Y.P.W. and X.D.P. reviewed the manuscript. All authors provided suggestions for manuscript revisions, reviewed the final draft, and agreed to submit it for publication.

## FUNDING INFORMATION

This work was supported by grants from Joint Funds for Innovation of Science and Technology, Fujian Province (No. 2021Y9037); the Financial Special Project of Fujian Province (No. 2021XH009); and the National Clinical Key Special Subject of China.

## CONFLICT OF INTEREST STATEMENT

No conflicts of interest exist.

## PATIENT CONSENT STATEMENT

All participants provided written informed consent at the time of study registration and data collection. Informed consent for participants with severe cognitive impairment was obtained from their guardians.

## Supporting information


Table S1.
Table S2.
Table S3.
Table S4.
Table S5.
Table S6.

## Data Availability

The data that support the findings of this study are available from the corresponding author upon reasonable request.
