# Supplementary material for: Brain white matter changes and their associations with non‐motor dysfunction in orthostatic hypotension in α‐synucleinopathy: A NODDI study
Source: CNS Neurosci Ther. 2024 Apr 14;30(4):e14712. doi: 10.1111/cns.14712 (PMC11016347; doi:10.1111/cns.14712)
Supplement: Supplementary file 1 — Table S1. Table S2. Table S3. Table S4. Table S5. Table S6. [file CNS-30-e14712-s001.docx]

**Supplementary table 1. TBSS analysis results between α-OH and α-NOH groups based on FISO parameters**

| **Cluster index** | **Regions & proportion, %** | **Cluster voxels** | **MNI-Peak coordinates** | | | ***P* - value** |
| --- | --- | --- | --- | --- | --- | --- |
|  |  |  | **X (mm)** | **Y (mm)** | **Z (mm)** |  |
| 1 | Anterior thalamic radiation L^a^:1.53742 | 62741 | 35 | -58 | -8 | 0.024 |
|  | Anterior thalamic radiation R^b^:1.02502 |  |  |  |  |  |
|  | Corticospinal tract L:0.808116 |  |  |  |  |  |
|  | Corticospinal tract R:0.840535 |  |  |  |  |  |
|  | Cingulum (cingulate gyrus) L:0.158238 |  |  |  |  |  |
|  | Cingulum (cingulate gyrus) R:0.141008 |  |  |  |  |  |
|  | Cingulum (hippocampus) L:0.0558805 |  |  |  |  |  |
|  | Cingulum (hippocampus) R:0.118455 |  |  |  |  |  |
|  | Forceps major:0.932229 |  |  |  |  |  |
|  | Forceps minor:2.00792 |  |  |  |  |  |
|  | Inferior fronto-occipital fasciculus L:1.67624 |  |  |  |  |  |
|  | Inferior fronto-occipital fasciculus R:1.90542 |  |  |  |  |  |
|  | Inferior longitudinal fasciculus L:1.43527 |  |  |  |  |  |
|  | Inferior longitudinal fasciculus R:1.13777 |  |  |  |  |  |
|  | Superior longitudinal fasciculus L:1.86105 |  |  |  |  |  |
|  | Superior longitudinal fasciculus R:1.52717 |  |  |  |  |  |
|  | Uncinate fasciculus L:0.539567 |  |  |  |  |  |
|  | Uncinate fasciculus R:0.273362 |  |  |  |  |  |
|  | Superior longitudinal fasciculus (temporal part) L:0.813774 |  |  |  |  |  |
|  | Superior longitudinal fasciculus (temporal part) R:0.54191 |  |  |  |  |  |
| a: L, left-lateralized fiber. | |  |  |  |  |  |
| b: R, right-lateralized fiber. | |  |  |  |  |  |

**Supplementary table 2. TBSS analysis results between α-OH and control groups based on NDI parameters**

| **Cluster index** | **Regions & proportion, %** | **Cluster voxels** | **MNI-Peak coordinates** | | | ***P* - value** |
| --- | --- | --- | --- | --- | --- | --- |
|  |  |  | **X (mm)** | **Y (mm)** | **Z (mm)** |  |
| 2 | Anterior thalamic radiation L^a^:2.06982 | 30679 | -11 | 18 | -12 | 0.008 |
|  | Anterior thalamic radiation R^b^:0.00176016 |  |  |  |  |  |
|  | Corticospinal tract L:0.597673 |  |  |  |  |  |
|  | Cingulum (cingulate gyrus) L:0.333094 |  |  |  |  |  |
|  | Cingulum (hippocampus) L:0.0847485 |  |  |  |  |  |
|  | Cingulum (hippocampus) R:9.77868e-05 |  |  |  |  |  |
|  | Forceps major:0.816878 |  |  |  |  |  |
|  | Forceps minor:2.81241 |  |  |  |  |  |
|  | Inferior fronto-occipital fasciculus L:2.96353 |  |  |  |  |  |
|  | Inferior longitudinal fasciculus L:2.49526 |  |  |  |  |  |
|  | Superior longitudinal fasciculus L:3.54826 |  |  |  |  |  |
|  | Superior longitudinal fasciculus R:0.000684507 |  |  |  |  |  |
|  | Uncinate fasciculus L:1.41025 |  |  |  |  |  |
|  | Uncinate fasciculus R:0.000195574 |  |  |  |  |  |
|  | Superior longitudinal fasciculus (temporal part) L:1.64158 |  |  |  |  |  |
|  | Superior longitudinal fasciculus (temporal part) R:0.00166237 |  |  |  |  |  |
| 1 | Anterior thalamic radiation L:0.00134354 | 22329 | 44 | 32 | 5 | 0.014 |
|  | Anterior thalamic radiation R:1.94514 |  |  |  |  |  |
|  | Corticospinal tract L:0.00107484 |  |  |  |  |  |
|  | Corticospinal tract R:0.0917193 |  |  |  |  |  |
|  | Cingulum (cingulate gyrus) R:0.0651619 |  |  |  |  |  |
|  | Cingulum (hippocampus) L:0.000268709 |  |  |  |  |  |
| 1 | Cingulum (hippocampus) R:0.0395898 | 22329 | 44 | 32 | 5 | 0.014 |
|  | Forceps major:0.759819 |  |  |  |  |  |
|  | Forceps minor:2.72583 |  |  |  |  |  |
|  | Inferior fronto-occipital fasciculus R:3.3701 |  |  |  |  |  |
|  | Inferior longitudinal fasciculus R:1.95109 |  |  |  |  |  |
|  | Superior longitudinal fasciculus L:0.000134354 |  |  |  |  |  |
|  | Superior longitudinal fasciculus R:2.23306 |  |  |  |  |  |
|  | Uncinate fasciculus R:0.926553 |  |  |  |  |  |
|  | Superior longitudinal fasciculus (temporal part) L:0.000134354 |  |  |  |  |  |
|  | Superior longitudinal fasciculus (temporal part) R:0.884679 |  |  |  |  |  |
| a: L, left-lateralized fiber. | |  |  |  |  |  |
| b: R, right-lateralized fiber. | |  |  |  |  |  |

**Supplementary table 3. TBSS analysis results between α-OH and control groups based on ODI parameters**

| **Cluster index** | **Regions & proportion, %** | **Cluster voxels** | **MNI-Peak coordinates** | | | ***P* - value** |
| --- | --- | --- | --- | --- | --- | --- |
|  |  |  | **X (mm)** | **Y (mm)** | **Z (mm)** |  |
| 7 | Anterior thalamic radiation R^a^:4.68518 | 3043 | 27 | -9 | 21 | 0.023 |
|  | Corticospinal tract L^b^:0.0266185 |  |  |  |  |  |
|  | Corticospinal tract R:6.21295 |  |  |  |  |  |
|  | Cingulum (cingulate gyrus) R:0.0414065 |  |  |  |  |  |
|  | Forceps major:0.000985869 |  |  |  |  |  |
|  | Forceps minor:0.0354913 |  |  |  |  |  |
|  | Inferior fronto-occipital fasciculus R:2.9001 |  |  |  |  |  |
|  | Inferior longitudinal fasciculus R:0.695695 |  |  |  |  |  |
|  | Superior longitudinal fasciculus R:8.39402 |  |  |  |  |  |
|  | Uncinate fasciculus R:0.022675 |  |  |  |  |  |
|  | Superior longitudinal fasciculus (temporal part) R:4.42261 |  |  |  |  |  |
| 6 | Anterior thalamic radiation L:0.126316 | 1140 | -43 | -33 | -12 | 0.038 |
|  | Cingulum (hippocampus) L:0.00263158 |  |  |  |  |  |
|  | Forceps minor:0.0236842 |  |  |  |  |  |
|  | Inferior fronto-occipital fasciculus L:8.03772 |  |  |  |  |  |
|  | Inferior longitudinal fasciculus L:16.3175 |  |  |  |  |  |
|  | Superior longitudinal fasciculus L:15.4211 |  |  |  |  |  |
|  | Uncinate fasciculus L:0.0526316 |  |  |  |  |  |
|  | Superior longitudinal fasciculus (temporal part) L:11.3184 |  |  |  |  |  |
| 5 | Anterior thalamic radiation L:0.469939 | 815 | -27 | -18 | 16 | 0.035 |
|  | Corticospinal tract L:15.3325 |  |  |  |  |  |
|  | Inferior fronto-occipital fasciculus L:0.0539877 |  |  |  |  |  |
| 5 | Inferior longitudinal fasciculus L:0.00368098 | 815 | -27 | -18 | 16 | 0.035 |
|  | Superior longitudinal fasciculus L:1.62822 |  |  |  |  |  |
|  | Uncinate fasciculus L:0.0736196 |  |  |  |  |  |
|  | Superior longitudinal fasciculus (temporal part) L:0.366871 |  |  |  |  |  |
| 4 | Inferior fronto-occipital fasciculus L:0.0700935 | 214 | -34 | -7 | 22 | 0.046 |
|  | Superior longitudinal fasciculus L:35.8598 |  |  |  |  |  |
|  | Superior longitudinal fasciculus (temporal part) L:18.0935 |  |  |  |  |  |
| 3 | Superior longitudinal fasciculus L:84.3393 | 56 | -37 | -28 | 30 | 0.049 |
|  | Superior longitudinal fasciculus (temporal part) L:41.0714 |  |  |  |  |  |
| 2 | Superior longitudinal fasciculus L:57.25 | 12 | -44 | -15 | 27 | 0.05 |
|  | Superior longitudinal fasciculus (temporal part) L:24.3333 |  |  |  |  |  |
| 1 | Superior longitudinal fasciculus L:0.9 | 10 | -29 | -33 | 15 | 0.05 |
| a: R, right-lateralized fiber. | |  |  |  |  |  |
| b: L, left-lateralized fiber. | |  |  |  |  |  |

**Supplementary table 4. TBSS analysis results between α-OH and control groups based on FISO parameters**

| **Cluster index** | **Regions & proportion, %** | **Cluster voxels** | **MNI-Peak coordinates** | | | ***P* - value** |
| --- | --- | --- | --- | --- | --- | --- |
|  |  |  | **X (mm)** | **Y (mm)** | **Z (mm)** |  |
| 1 | Anterior thalamic radiation L^a^:1.17663 | 122519 | 47 | -13 | -26 | 0.001 |
|  | Anterior thalamic radiation R^b^:0.972861 |  |  |  |  |  |
|  | Corticospinal tract L:0.777896 |  |  |  |  |  |
|  | Corticospinal tract R:0.770003 |  |  |  |  |  |
|  | Cingulum (cingulate gyrus) L:0.307724 |  |  |  |  |  |
|  | Cingulum (cingulate gyrus) R:0.142925 |  |  |  |  |  |
|  | Cingulum (hippocampus) L:0.112236 |  |  |  |  |  |
|  | Cingulum (hippocampus) R:0.124454 |  |  |  |  |  |
|  | Forceps major:0.722549 |  |  |  |  |  |
|  | Forceps minor:2.05113 |  |  |  |  |  |
|  | Inferior fronto-occipital fasciculus L:1.29204 |  |  |  |  |  |
|  | Inferior fronto-occipital fasciculus R:1.36443 |  |  |  |  |  |
|  | Inferior longitudinal fasciculus L:1.14435 |  |  |  |  |  |
|  | Inferior longitudinal fasciculus R:0.881937 |  |  |  |  |  |
|  | Superior longitudinal fasciculus L:1.38103 |  |  |  |  |  |
|  | Superior longitudinal fasciculus R:1.20665 |  |  |  |  |  |
|  | Uncinate fasciculus L:0.464818 |  |  |  |  |  |
|  | Uncinate fasciculus R:0.246868 |  |  |  |  |  |
|  | Superior longitudinal fasciculus (temporal part) L:0.623675 |  |  |  |  |  |
|  | Superior longitudinal fasciculus (temporal part) R:0.443115 |  |  |  |  |  |
| a: L, left-lateralized fiber. | |  |  |  |  |  |
| b: R, right-lateralized fiber. | |  |  |  |  |  |

**Supplementary table 5. TBSS analysis results between α-NOH and control groups based on ODI parameters**

| **Cluster index** | **Regions & proportion, %** | **Cluster voxels** | **MNI-Peak coordinates** | | | ***P* - value** |
| --- | --- | --- | --- | --- | --- | --- |
|  |  |  | **X (mm)** | **Y (mm)** | **Z (mm)** |  |
| 5 | Anterior thalamic radiation L^a^:2.47179 | 12834 | 14 | -28 | 29 | 0.02 |
|  | Anterior thalamic radiation R^b^:1.65295 |  |  |  |  |  |
|  | Corticospinal tract L:0.567087 |  |  |  |  |  |
|  | Corticospinal tract R:0.982313 |  |  |  |  |  |
|  | Cingulum (cingulate gyrus) L:1.39707 |  |  |  |  |  |
|  | Cingulum (cingulate gyrus) R:0.934393 |  |  |  |  |  |
|  | Cingulum (hippocampus) L:0.00701262 |  |  |  |  |  |
|  | Cingulum (hippocampus) R:0.0252454 |  |  |  |  |  |
|  | Forceps major:0.345956 |  |  |  |  |  |
|  | Forceps minor:5.08992 |  |  |  |  |  |
|  | Inferior fronto-occipital fasciculus L:1.76773 |  |  |  |  |  |
|  | Inferior fronto-occipital fasciculus R:2.18326 |  |  |  |  |  |
|  | Inferior longitudinal fasciculus L:3.15132 |  |  |  |  |  |
|  | Inferior longitudinal fasciculus R:0.653265 |  |  |  |  |  |
|  | Superior longitudinal fasciculus L:3.8504 |  |  |  |  |  |
|  | Superior longitudinal fasciculus R:0.241078 |  |  |  |  |  |
|  | Uncinate fasciculus L:0.0993455 |  |  |  |  |  |
|  | Uncinate fasciculus R:0.714275 |  |  |  |  |  |
|  | Superior longitudinal fasciculus (temporal part) L:2.22433 |  |  |  |  |  |
|  | Superior longitudinal fasciculus (temporal part) R:0.142278 |  |  |  |  |  |
| 4 | Cingulum (cingulate gyrus) L:3.03774 | 53 | -9 | -58 | 20 | 0.049 |
|  | Cingulum (hippocampus) L:0.0566038 |  |  |  |  |  |
| 3 | Inferior fronto-occipital fasciculus L:13.8286 | 35 | -32 | 4 | -10 | 0.05 |
|  | Superior longitudinal fasciculus L:0.171429 |  |  |  |  |  |
|  | Uncinate fasciculus L:43.9714 |  |  |  |  |  |
|  | Superior longitudinal fasciculus (temporal part) L:0.428571 |  |  |  |  |  |
| 2 | Cingulum (cingulate gyrus) L:3.92593 | 27 | -7 | -56 | 11 | 0.05 |
|  | Cingulum (hippocampus) L:0.444444 |  |  |  |  |  |
| 1 | Anterior thalamic radiation R:2.3 | 10 | 27 | 30 | 21 | 0.05 |
|  | Inferior fronto-occipital fasciculus R:0.9 |  |  |  |  |  |
| a: L, left-lateralized fiber. | |  |  |  |  |  |
| b: R, right-lateralized fiber. | |  |  |  |  |  |

**Supplementary table 6. TBSS analysis results between α-NOH and control groups based on FISO parameters**

| **Cluster index** | **Regions & proportion, %** | **Cluster voxels** | **MNI-Peak coordinates** | | | ***P* - value** |
| --- | --- | --- | --- | --- | --- | --- |
|  |  |  | **X (mm)** | **Y (mm)** | **Z (mm)** |  |
| 1 | Anterior thalamic radiation L^a^:1.13851 | 89696 | -42 | -25 | 30 | 0.016 |
|  | Anterior thalamic radiation R^b^:0.934456 |  |  |  |  |  |
|  | Corticospinal tract L:0.613461 |  |  |  |  |  |
|  | Corticospinal tract R:0.607797 |  |  |  |  |  |
|  | Cingulum (cingulate gyrus) L:0.391534 |  |  |  |  |  |
|  | Cingulum (cingulate gyrus) R:0.189652 |  |  |  |  |  |
|  | Cingulum (hippocampus) L:0.0197445 |  |  |  |  |  |
|  | Cingulum (hippocampus) R:0.147833 |  |  |  |  |  |
|  | Forceps major:0.726721 |  |  |  |  |  |
|  | Forceps minor:2.52125 |  |  |  |  |  |
|  | Inferior fronto-occipital fasciculus L:1.54418 |  |  |  |  |  |
|  | Inferior fronto-occipital fasciculus R:1.67929 |  |  |  |  |  |
|  | Inferior longitudinal fasciculus L:1.38236 |  |  |  |  |  |
|  | Inferior longitudinal fasciculus R:1.0714 |  |  |  |  |  |
|  | Superior longitudinal fasciculus L:1.72056 |  |  |  |  |  |
|  | Superior longitudinal fasciculus R:1.54248 |  |  |  |  |  |
|  | Uncinate fasciculus L:0.571051 |  |  |  |  |  |
|  | Uncinate fasciculus R:0.311051 |  |  |  |  |  |
|  | Superior longitudinal fasciculus (temporal part) L:0.785999 |  |  |  |  |  |
|  | Superior longitudinal fasciculus (temporal part) R:0.582702 |  |  |  |  |  |

a: L, left-lateralized fiber.

b: R, right-lateralized fiber.
